# Elasto-Plastic Mechanical Modeling of Fused Deposition 3D Printing Materials

**DOI:** 10.3390/polym15010234

**Published:** 2023-01-02

**Authors:** Francesco Bandinelli, Lorenzo Peroni, Alberto Morena

**Affiliations:** Department of Mechanical and Aerospace Engineering, Politecnico di Torino, Corso Duca degli Abruzzi, 24, 10129 Turin, Italy

**Keywords:** fused deposition modeling, additive manufacturing, carbon–nylon, tensile tests, anisotropic elasto-plastic behavior, Hill criterion, Voce hardening law, material model identification, LS-DYNA

## Abstract

Fused deposition modeling (FDM) is one of the most common 3D printing technologies and is becoming a well-established production method. Short fiber-reinforced polymers represent a new class of printing materials that enhance the mechanical properties of final components, thus informing an interesting subject of analysis for this study. FDM-printed parts are characterized by a strong anisotropy, so their behavior should be analyzed accordingly. The authors proposed a modeling strategy based on a transversely isotropic behavior hypothesis, representing material behaviors associated with an elasticity matrix in relation to the elastic field and a combination of Hill’s yield criterion and Voce’s isotropic hardening law for the plastic field. Material properties of materials were experimentally identified through tensile tests on dog bone specimens printed with different orientations in space. The numerical model was then calibrated using the finite element software LS-DYNA and the optimization software LS-OPT. An agreement between numerical and experimental results showed the robustness of the modeling strategy proposed to describe the stress behaviors of printed materials until a maximum load is reached, while strain behaviors have yet to be correctly defined due to the difficulties associated with evaluating an equivalent deformation.

## 1. Introduction

Over the last few decades, additive manufacturing technologies evolved substantially, offering a valid alternative to common production techniques. Additive manufacturing started as a rapid prototyping tool and evolved to become a unique production method used to fabricate complex-shaped parts. This work will focus on fused deposition modeling (FDM), one of the most common additive manufacturing techniques [1]. This method seeks to build the final component by creating a sequence of layers, deposing a fused material filament, and stacking them along the printing direction. Using a heated nozzle, the material is extruded and deposited on the printing plane in a semi-molten state, so the final structure is composed of numerous filaments arranged in different directions following the building strategy. Furthermore, the adhesion between different layers is affected by the non-complete junction of filaments. Printed materials have mechanical properties that strongly depend on these particular features, known as raster angles and layer adhesion, in turn complexifying the characterization of their behavior. The difference between the mechanical properties in the building direction and those in the printing plane makes the material extremely anisotropic, so it is necessary to describe its behavior in light of this fundamental aspect. In addition to the raster angle, many other parameters play an important role in defining the mechanical properties of FDM materials, such as the layer height, filament width, nozzle diameter, nozzle temperature, bed temperature, and printing speed [2,3,4,5]. Moreover, from the different combination of these inputs, a wide variety of properties of the final component can be achieved. However, the printing parameters are related to printing process options and thus will not be analyzed in this paper. Furthermore, the results of the printing process are highly dependent on pristine filament conditions (such as the stocking humidity and temperature), making it rather difficult to obtain the same mechanical characteristics in some cases, even when using the same printing parameters. For this reason, the authors observed a considerable dispersion of results between the different batches of specimens and decided to focus their analysis on each batch separately. In particular, experimental results showed a maximum standard deviation of 15% for Young’s modulus, a maximum of 7% for the yield stress, and a maximum of 7% for the ultimate strength for the same specimen orientation through different batches. The modeling methodology that will be proposed does not lose generality, but highlights the fact that mechanical properties evaluated from a single print have to be considered as valid for that batch only, when the process control is not completely achievable. Thus, when operating on real components, the correct material properties will be evaluated.

Usually, filaments used for FDM are made out of isotropic materials, but the result of the printing process always leads to the creation of highly oriented materials due to the deposition strategy and adhesion problems between layers. In recent years, significant progress was made in the ability to print new materials, such as short fiber-reinforced polymers. The addition of particles and fibers inside the matrix promotes the achievement of higher mechanical properties and makes FDM products much more interesting from the point of view of performances. In particular, this work will focus on the characterization of a polyamide reinforced with short carbon fibers, which is extremely attractive given their light weight and high strength.

Many research studies have focused on the characterization of the mechanical properties of FDM parts using tensile tests [6,7,8,9]. The raster angle of the specimens was varied to investigate the dependence from the direction tested of elastic moduli [10,11,12,13], tensile elastic limits [7,10,14], and tensile ultimate strengths [7]. The results show a great difference between the mechanical properties in various directions, displaying the strong anisotropy that characterizes FDM-printed parts.

Finite element analysis represents a very important tool for mechanical design and verification; however, it has not been fully developed for this type of component. Many research works have attempted to model the behavior of FDM parts, mainly focusing on elastic properties [11,15] and ultimate tensile strength [16,17,18], achieving interesting results. The plastic behavior of FDM materials has also been investigated and some works proposed strategies to describe it [10,19]; however, this aspect is particularly difficult to model due to the lack of understanding of this phenomenon. Hill formalized a yield criterion for anisotropic materials, taking metal rolled sheets as a reference [20]. Some authors proposed using Hill’s anisotropic yield criterion to model the transition from elastic fields to plastic fields and the successive plastic deformation through an isotropic hardening rule [10,19]. The results showed that numerical predictions can be quite accurate and can satisfactorily represent the material behavior. However, only a few attempts were made to use finite element analysis as a design or verification tool [10], highlighting the need to improve the understanding of material models that are capable of representing FDM parts, especially in terms of their plastic and failure behavior.

Given the printing strategy of the FDM process, the differences in mechanical properties in the printing plane can be neglected [11,15,17,18] or can be seen as equivalent those in the vertical plane [19]; hence, the material can be considered as transversely isotropic.

In this paper, the authors propose a methodology to model the elastic and the plastic behavior of components printed by fused deposition modeling, using the example of a short fiber-reinforced polymer. In this sense, specimens with different angles with respect to the printing plane were built and tested. From the experimental data, a transversely isotropic stiffness matrix was defined to model the elastic behavior, while a combination of the anisotropic yield criterion proposed by Hill and Voce’s isotropic hardening law was used in order to describe the plastic behavior.

## 2. Modeling Methodology

**Figure 1:**
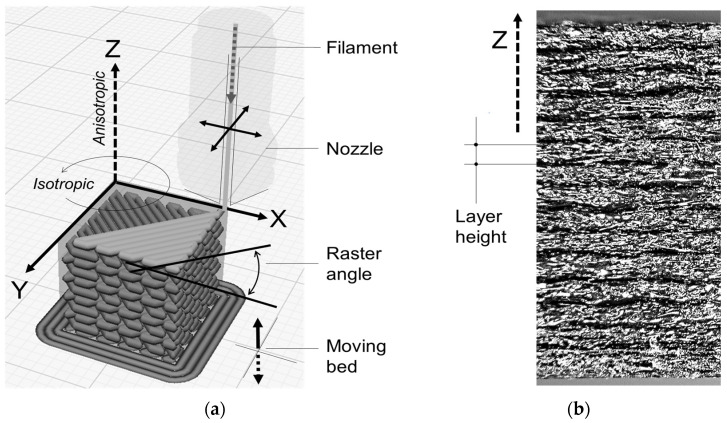
Scheme of the FDM process. (**a**) Details of the external surface of a printed component (**b**) made with a carbon fiber-reinforced polyamide filament.

The printing process of FDM parts is based on a deposition strategy that adopts the printer reference system which defines the nozzle movement. The material is thus characterized by three main directions which identify the mechanical properties. The printing growth direction will be indicated as the Z direction, and those in the printing plane will be indicated as X and Y directions, as shown in Figure 1. The printing strategy strongly affects material properties, so the raster angle and the stacking sequence of the layers are relevant parameters for the mechanical performance of the final component. In the present work, the authors decided to alternate +45° and −45° raster angles, as shown in Figure 1, attributing the transversely isotropic hypothesis to this choice [11,15,17,18].

Conducting analyses of 3D-printed structures requires the knowledge of constitutive equations which can model material behaviors with a continuum mechanics approach. Due to the actual available computing power, it is difficult, if not impossible, to realize numerical models that can simulate entire components with a microstructural level of detail. Microscale models can be effectively used only on limited material portions to obtain homogenized model characteristics, that are useful when implementing mechanical analyses on real components, starting from the properties of mesostructures [21,22,23]. However, even in these cases, it is possible and probably needed to consider material anisotropy.

Plastic material filaments are made out of a polymeric matrix that can be reinforced using short fibers. When this type of filament is printed using the FDM technique, matrix polymeric chains are deposited on the printing plane following the nozzle path. The microstructure is composed of a matrix in which the reinforcement fibers are dispersed. The final result is a highly inhomogeneous structure that does not present the same crystalline structure as metals or their alloys, in which dislocations can be found, but can be conveniently modeled as a series of reversible or elastic behaviors and irreversible or plastic behaviors. Even if the same physics does not hold for the definition of the two deformation behaviors, from a purely phenomenological point of view, the reversible part can be treated with reference to a constitutive model based on the elasticity matrix (or equivalently its inverse), while the irreversible part can be treated with reference to a combination of a yield criterion, a plastic flow rule, and a hardening law.

The first will describe the transition between the elastic and plastic behaviors in all the possible multiaxial stress combinations. The second will define the relation between the plastic strain rate and the stress tensor, while the last will rule the yield criterion evolution and yield surface evolution through plastic deformation and the other variables involved (i.e., strain rate and temperature).

### 2.1. Elastic behavior

For what the elastic behavior is concerned, given the stratification obtained with the FDM technique, a compliance matrix can be defined. With reference to the orthogonal system identified by the printing directions, the general orthotropic elastic behavior is based on the definition of the following compliance matrix expressed by Equation (1).

(1) $$\left\{\begin{array}{c} \begin{array}{c} \varepsilon_{xx}\\ \varepsilon_{yy}\\ \varepsilon_{zz} \end{array}\\ \begin{array}{c} \gamma_{xy}\\ \gamma_{yz}\\ \gamma_{xz} \end{array} \end{array}\right\}=\left[\begin{array}{cc} \begin{array}{ccc} 1/E_{xx} & -\nu_{xy}/E_{yy} & -\nu_{xz}/E_{zz}\\ -\nu_{yx}/E_{xx} & 1/E_{yy} & -\nu_{yz}/E_{zz}\\ -\nu_{zx}/E_{xx} & -\nu_{zy}/E_{yy} & 1/E_{zz} \end{array} & \\ & \begin{array}{ccc} 1/G_{xy} & & \\ & 1/G_{yz} & \\ & & 1/G_{xz} \end{array} \end{array}\right]\left\{\begin{array}{c} \begin{array}{c} \sigma_{xx}\\ \sigma_{yy}\\ \sigma_{zz} \end{array}\\ \begin{array}{c} \tau_{xy}\\ \tau_{yz}\\ \tau_{xz} \end{array} \end{array}\right\}$$

Indeed, the matrix has to fulfill the symmetry condition given by (2)

(2) $$\frac{\nu_{ij}}{E_{ii}}=\frac{\nu_{ji}}{E_{jj}}$$

From Equations (1) and (2), it is clear that the number of elastic constants to be defined is 9, which comes from the 12 total elastic constants definable for an orthotropic material minus the 3 symmetry conditions. After introducing the engineering assumption of transversely isotropic material, the elastic moduli in the printing plane are equivalent; hence, $E_{xx}=E_{yy}$, $G_{xz}=G_{yz}$ and $\nu_{zx}=\nu_{zy}$. The assumption of isotropic behavior in the XY plane also allows the following relation to be introduced, linking the shear elastic modulus to Young’s modulus by means of Poisson’s coefficient (3):

(3) $$G_{xy}=\frac{E_{xx}}{2\left(1+\nu_{xy}\right)}$$

The newly introduced equations lower the number of independent elastic constants to 5, alongside the elastic moduli in the Z and X (or equivalently Y) directions $E_{zz}$ and $E_{xx}$, Poisson’s coefficients $\nu_{xy}$ and $\nu_{zx}$, and the shear elastic modulus $G_{xz}$ (or equivalently $G_{yz}$). All these elastic constants can be easily obtained by means of appropriate tensile tests that are usually performed for composite materials [24].

**Figure 2:**
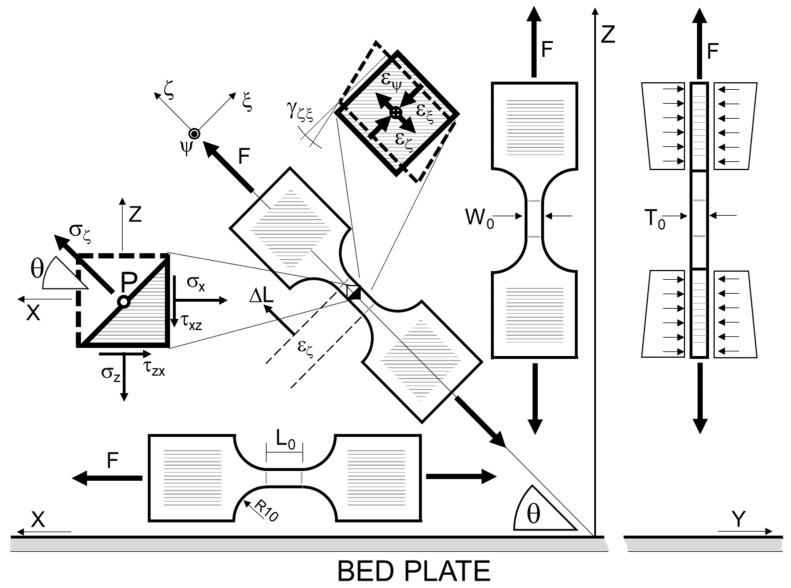
Representation of specimens with definitions of the printer reference system (XYZ) and testing reference system (ζψξ).

In order to completely characterize the elastic compliance matrix, a batch of specimens, retaining a longitudinal direction with different angles relating to the printing plane, can be designed, as represented in Figure 2. This allows material properties with varying material directions to be investigated and characterized, and so to evaluate anisotropy, e.g., by assuming θ angles equal to 0° (on-edge specimen), 45°, and 90° (vertical specimen).

Particular attention shall be paid to the difference between the reference system of the main material directions (indicated as XYZ) and that of the tensile test (indicated as ζψξ). In fact, when anisotropic materials are subjected to loads in directions that differ from those of the anisotropy, the stiffness matrix has to be rotated accordingly for it to become fully populated. Thus, stiffness terms that link normal stresses to shear deformations are created, so even a uniaxial stress state can result in the birth of a shear strain. This effect causes the directions of the principal strains to be different from those of the principal stresses. In the case of a tensile test, the latter stresses are aligned with the testing directions, while the former can be visibly discordant.

### 2.2. Plastic Behavior

Many previous works on FDM-printed materials highlighted that the material generally exhibits an orthotropic plastic behavior besides its elastic orthotropic one [8,10,19,25]. By dealing with metallic materials, the orthotropic yield condition could be described with Hill’s criterion. Thus, it is reasonable to imagine that this formulation could also be adapted to model fiber-reinforced polymeric materials produced by the FDM technique. Hill’s yield criterion is based on a formulation of an anisotropic yield surface, so it is obviously different from the von Mises’ cylinder in the principal stress coordinate system and it needs a yield function that involves the whole stress tensor. In the case of printed materials, the most coherent choice is to use the printer reference system because it defines orthotropic characteristics. In this reference system, the general orthotropic material exhibits different normal yield stresses depending on the uniaxial stress condition considered in the three directions (X, Y, and Z), as well as the shear yield stresses that are not directly linked to the latter or even between them. Until now, no assumption has been made to use this formulation when treating printed materials, since Hill’s yield criterion is formulated for general anisotropic materials. Even in the case of porous polymers, the same approach could be used to consider the material as homogenous.

The orthotropic plastic behavior of FDM-printed materials will then be described by Hill’s yield criterion, which is stated in Equation (4).

(4) $$F{\left(\sigma_{xx}-\sigma_{yy}\right)}^{2}+G{\left(\sigma_{yy}-\sigma_{zz}\right)}^{2}+H{\left(\sigma_{zz}-\sigma_{xx}\right)}^{2}+2L\tau_{xy}^{2}+2M\tau_{yz}^{2}+2N\tau_{xz}^{2}{}^{}=1$$

Hill’s plastic potential is a generalization of the well-known von Mises’ yield criterion, which was developed to characterize the anisotropy of metal rolled sheets. Hill’s parameters *F*, *G*, *H*, *L*, *M*, and *N* can be evaluated from the yield stresses in the material principal anisotropy axes using the relations from [20], as reported in Equation (5).

(5) $$\frac{1}{Y_{xx}^{2}}=G+H\;\frac{1}{Y_{yy}^{2}}=H+F\;\frac{1}{Y_{zz}^{2}}=F+G\;2F=\frac{1}{Y_{yy}^{2}}+\frac{1}{Y_{zz}^{2}}-\frac{1}{Y_{xx}^{2}}\;2G=\frac{1}{Y_{zz}^{2}}+\frac{1}{Y_{xx}^{2}}-\frac{1}{Y_{yy}^{2}}\;2H=\frac{1}{Y_{xx}^{2}}+\frac{1}{Y_{yy}^{2}}-\frac{1}{Y_{zz}^{2}}\;2L=\frac{1}{Y_{yz}^{2}}\;2M=\frac{1}{Y_{zx}^{2}}\;2N=\frac{1}{Y_{xy}^{2}}$$

where $Y_{ij\;}$ is the yield stress in the *ij* direction. It should be noted that the yield strength in *X* and *Y* directions must be the same according to the transversely isotropic hypothesis, so *Y_xx_* and *Y_yy_* are to be considered equal.

It is well known that a yield criterion only establishes the transition between the elastic and plastic behaviors. Once the yield is reached, the material remains in that condition until a stress reduction brings it back to the elastic regime. During its permanence in the plastic field, the stress tensor can only change its values moving on the yield surface. To describe the phase of the plastic flow, which represents the evolution of the irreversible deformations process, it is necessary to combine the yield criterion with a flow rule. By always keeping metallic materials as a reference, the combination of Druker’s postulate and the volume conservation helps to define the Levy–Mises flow rule.

After applying Hill’s yield criterion to the Levy–Mises flow rule, the relations between the strain increments and the stress components during plastic deformation can be generalized for anisotropic components, as reported in Equation (6).

(6) $$d\varepsilon_{xx}=d\lambda \;\left[H\left(\sigma_{xx}-\sigma_{yy}\right)+G\left(\sigma_{xx}-\sigma_{zz}\right)\right]\;d\varepsilon_{yz}=d\lambda \;L\sigma_{yz}\;d\varepsilon_{yy}=d\lambda \;\left[F\left(\sigma_{yy}-\sigma_{zz}\right)+H\left(\sigma_{yy}-\sigma_{xx}\right)\right]\;d\varepsilon_{zx}=d\lambda \;M\sigma_{zx}\;d\varepsilon_{zz}=d\lambda \;\left[G\left(\sigma_{zz}-\sigma_{xx}\right)+F\left(\sigma_{zz}-\sigma_{yy}\right)\right]\;d\varepsilon_{xy}=d\lambda \;N\sigma_{xy}$$

As anticipated, the theory can be formulated for metals in which the plastic deformation is based on the dislocation motion, causing the volume change to be negligible, as clearly seen from the fact that the $d\varepsilon_{xx}+d\varepsilon_{yy}+d\varepsilon_{zz}=0$ relation is respected. However, FDM-printed plastic materials are generally characterized by significant porosity which might make the zero-volume variation a strong approximation; thus, the previous statement will need to be accurately evaluated. Experimental results highlight that printed materials also exhibit a post-yielding hardening behavior, so they tend to increase their resistance and plastic flow stress with rising strain values. In metals, this behavior is due to the blocking mechanisms of dislocation movements, while in polymeric material it is caused by a progressive reduction in the reciprocal mobility of polymeric chains. The strain-induced crystallization of the polymeric matrix can effectively explain the hardening behavior of the material. This phenomenon occurs regardless of the material orientation and enhances mechanical properties in the testing direction, thus allowing us to assume an isotropic hardening law to describe the plastic behavior.

From a theoretical point of view, it is then necessary to combine the yield criterion and the plastic flow rule with a hardening law that is capable of increasing the size of the yield surface with rising deformation. It is also necessary to define a parameter which can rule this growth in relation to the strain tensor. This parameter is usually defined as the plastic equivalent strain and it is identified as a combination of strain tensor components, from which the elastic portion is subtracted. Given the generally strain-incremental nature of the plastic treatment, the equivalent plastic strain will be obtained by the time integration of plastic strain rates, using a definition that is coherent with the adopted yield criterion [26].

As already discussed, plasticity needs a hardening law $f{\left(\varepsilon_{eq}^{p},{\dot{\varepsilon }}_{eq}^{p}\right)}_{}$ to define the evolution of the yield surface with plastic strain and a strain rate. In general, Equation (4) can be rewritten as follows:

(7) $$F{\left(\sigma_{xx}-\sigma_{yy}\right)}^{2}+G{\left(\sigma_{yy}-\sigma_{zz}\right)}^{2}+H{\left(\sigma_{zz}-\sigma_{xx}\right)}^{2}+2L\tau_{xy}^{2}+2M\tau_{yz}^{2}+2N\tau_{xz}^{2}{}^{}=f{\left(\varepsilon_{eq}^{p},{\dot{\varepsilon }}_{eq}^{p}\right)}_{}$$

where $f{\left(\varepsilon_{eq}^{p},{\dot{\varepsilon }}_{eq}^{p}\right)}_{}=1$ for $\varepsilon_{eq}^{p}=0$. The fact that the curve needs to be given as a function of the equivalent plastic strain should not be underestimated. In fact, in order to evaluate the equivalent strain, the principal strains need to be known. However, in general, the principal strain directions do not coincide with the directions in which the component appears to be loaded or with the principal axes of anisotropy of the material, which is the reference system in which Hill’s relations have the form reported in Equations (4) to (7). Given the element represented in Figure 2 pulled along the ζ direction which is tilted by an angle θ with the direction X, it is generally true that the principal strain directions do not coincide with direction ζ, including both X and Z, which are the anisotropy principal directions of the material. In addition to this, the calculation of the strain increments from Equation (6) requires that Hill’s parameters are fixed; otherwise, relations between strain increments in the different material directions would be undefined.

In this sense, the correct approach would require the deformation exhibited by the element to be determined, the relative strain tensor to be built, the strain tensor to be rotated to align it to the principal anisotropic directions, the incremental deformation to be evaluated from the flow rule stated in Equation (6), the principal strains from the resulting strain tensor to be computed, and finally the equivalent strain to be defined. Indeed, several finite element (FE) software is specifically developed to carry out these mathematical operations and they can be opportunely coupled with optimization algorithms to run iteratively FE codes in order to find a solution.

One of the most used isotropic hardening laws is Voce’s law [27]; with this, by neglecting the strain-rate effects, the relation between the equivalent stress and the equivalent plastic strain can be defined by means of up to four coefficients, as reported in Equation (8).

(8) $$\sigma \left(\varepsilon_{eq}^{p}\right)=\sigma_{0}+Q_{r1}\left[\left(1-exp\left(-C_{r1}\varepsilon_{eq}^{p}\right)\right)]+Q_{r2}[\left(1-exp\left(-C_{r2}\varepsilon_{eq}^{p}\right)\right)\right]$$

In view of the description of plasticity modeling, it is clear that the specimens theorized in the previous section can also be used to completely define the plastic behavior of the material. From simple tensile tests performed with the aforementioned specimens, *F*, *G*, *H*, and normal yield stress are Hill’s parameters that can be easily identified, while shear stress parameters *L*, *M*, and *N* can be numerically defined by minimizing the difference between the experimental yield points and Hill’s yield surface. The parts printed by FDM are usually composed of thin walls; hence, the specimens should also have a relatively small thickness. In this case, the plane stress approximation is appropriate and the shear stress parameters need to be numerically lower to just one between *L*, *M*, and *N*.

Concerning the evaluation of Voce’s hardening law coefficients, given the complexity of building an equivalent plastic strain–stress curve from uniaxial tensile tests, the authors decided to develop an LS-DYNA finite element model coupled with an LS-OPT optimization solver in which single-shell elements with different loading conditions were considered. In this optimization procedure, Voce’s coefficients that gave the best agreement between the force–displacement curves coming from the experimental and numerical tensile tests were discovered.

It is interesting to highlight that Hill’s parameters, which can be evaluated for the yielding condition, also affect the whole plastic behavior from Equation (6). Hence, the best fitting can be obtained by also requesting Hill’s parameter to the solver; however, the increase in precision obtainable with this procedure is so negligible that it does not justify the increase in computational effort.

## 3. Materials and Methods

Due to the ever-increasing interest in the new class of FDM materials represented by fiber-reinforced plastics, especially for their high stiffness compared to non-reinforced ones, the authors decided to take carbon fiber-reinforced nylon as a reference material to describe the mechanical properties using a characterization method. Hence, the polymeric material from which the specimens were built was Nylforce Carbon produced by FiberForce (Treviso, Italy). It is made of a nylon matrix (PA) which is reinforced by short carbon fibers with a content up to 20% in weight. The reinforced composite combines high mechanical properties with excellent printability, strengthening the possibility of 3D-printing components to withstand severe loading, even in harsh thermal and chemical conditions.

**Figure 3:**
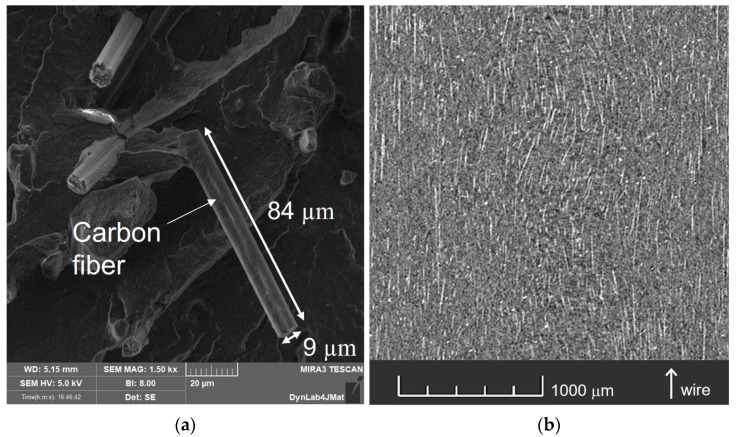
Electron microscope scan of the fracture surface of a broken wire (**a**) and micro-CT scan of the same wire (**b**) where carbon fibers are visible (white straight lines).

Figure 3 reports the scanning electron microscopy and micro-CT scan images of the wire. By analyzing the images, it appeared that carbon fibers have a preferential orientation given by the fabrication process (injection molding), so they were mainly oriented in the longitudinal direction of the wire. The fibers had a diameter of about 8–10 μm and a length of about 80–100 μm. When the wire was extruded during printing, it was reasonable to assume that this fiber distribution and orientation was preserved.

The properties of the material “as-printed” declared by the manufacturer are reported in Table 1.

Tensile tests were conducted according to the experimental procedure described by the ASTM D638 standard with small variations in specimen geometry to adapt it to the load capacity and gripping fixture of the testing machine, as well as to better exploit the camera resolution in tracking analyses.

For the present experimental campaign, the specimens were designed with a small gauge section of 5 mm × 5 mm, while the gauge length was 10 mm. This choice allowed all the specimens to be produced in the same print which guarantees the same printing conditions to have printing parameters, printing environments, and filament conditions for the whole batch, while also minimizing printing times and material consumption. The reduced gauge section was also chosen to guarantee the compatibility in terms of the maximum load and strain at failure with high-strain-rate systems, such as Hopkinson bars, for future work.

The 3D printer used was an Ultimaker S5 (Ultimaker B.V., Utrecht, NL) with a 0.6 mm nozzle. The print speed was fixed at 40 mm/s, the nozzle temperature was 275 °C, and the bed temperature was 70 °C, while the infill percentage was 100% and the raster angle was ±45°. The choice of raster angle and stacking sequence makes it reasonable to consider the printed components as transversely isotropic, where the directions of isotropy lie in the printing plane, as also shown in previous works [11,15,17,18]. This hypothesis helps to define the geometry of the specimen batch, given that the aim of the tests is to evaluate the anisotropy introduced by the printing process, focusing on the change in mechanical properties from the printing plane directions X and Y to the vertical direction Z. To properly capture this behavior, the specimens were printed in order to vary the test direction from the one lying in the printing plane to that coinciding with the vertical one. Thus, 7 dogbone specimens were printed in a single batch ranging the angle θ from 0° to 90° at constant steps of 15°. The ζ direction of the 0° specimen coincideswith the X direction, while the 90° one coincides with the Z direction. In addition, an extra specimen was added to each batch to capture the behavior in the isotropic plane XY; in fact, it was entirely printed while lying in the printing plane. This specimen, indicated as “PL”, allows Poisson’s coefficient *ν_xy_* to be measured and increases the number of specimens per batch to 8.

**Figure 4:**
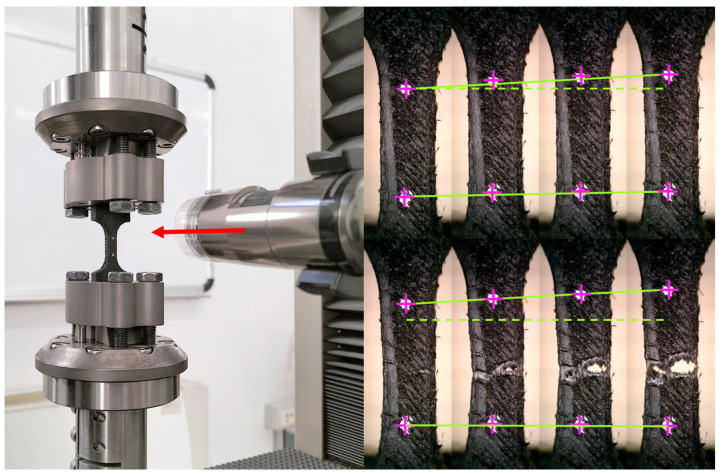
Experimental setup and scheme of the video tracking analysis.

The tensile tests were performed by means of the Zwick-Z05 (ZwickRoell GmbH & Co. KG, Ulm, Denmark) standard testing machine (shown in Figure 4) at a constant engineering strain rate of 0.01 s^−1^. The load cell installed on the machine guarantees an accuracy of ±0.5% (ISO 7500: accuracy grade 0.5) with a maximum measuring force of 5 kN. Each test was recorded with the high-resolution (2592 × 1944 pixels) Dino-Lite AD7013MT camera (AnMo Electronics Corporation, Taipei, Taiwan) with a magnification of 20× at a framerate of 8 fps so that the videos could be analyzed to evaluate strain evolution in time. Lightning was adapted by the polarizing filter of the camera.

In addition to standard tensile tests, cyclic loading and unloading tensile tests were also conducted to evaluate the linear elastic behavior deformation range, so that Young’s moduli of the various specimens could be calculated accordingly. These tests were conducted on the same type of specimens presented previously, with load steps of 100 N and unloading at each increase until failure, allowing the elastic behavior to be evaluated during the unloading phase and the stored plastic deformation.

## 4. Results and Discussion

### 4.1. Tensile Tests

Each tensile test video was processed using the digital image correlation software DICe [28] in order to execute tracking analyses. To obtain Young’s moduli of the specimens, cyclic loading and unloading tensile tests were conducted. This allowed the limits of the linear elastic behavior to be evaluated, which were found to be at around 30% of the maximum load across the various specimens. It is important to highlight that elastic moduli were determined in the direction of the test (direction ζ of Figure 2), which is generally different from the principal anisotropic directions, as discussed previously. In the particular cases of specimens oriented at 0° and 90°, the testing direction was aligned with X and Z axes, respectively, so their Young’s moduli coincides with those of the material main directions $E_{xx}$ and $E_{zz}$. Thus, Young’s moduli of the various specimens in the test direction were found and the results are reported in Table 2. The results show that 0° and PL specimens have a different Young’s modulus and a different tensile strength, even sharing the same orientation with respect to the printing direction.

This fact highlights the effects of the printing process, so the authors decided to only consider the PL specimen for Poisson’s coefficient evaluation, leaving Young’s modulus in the X direction to only be calculated on the 0° specimen data.

Tracking analyses were also used to evaluate Poisson’s coefficients. In particular, the PL specimen was used to estimate Poisson’s coefficients of the printing plane ($\nu_{xy}$), while the 90° specimen was used to make estimations of in vertical plane ($\nu_{zx}$ or equivalently $\nu_{zy}$). Once the Poisson coefficient in the isotropic plane is known, the shear modulus $G_{xy}$ can be computed from Equation (3). In the case of a tensile test, it is well known that the stress condition is uniaxial; hence, the shear modulus $G_{xz}$ can be found by Equation (9), which is generally used for composites [24].

(9) $$G_{xz}={\left(\frac{1}{E_{\zeta }\;cos^{2}\theta \;sin^{2}\theta }-\frac{1}{E_{zz}\;tg^{2}\theta }-\frac{tg^{2}\theta }{E_{xx}}+\frac{2\nu_{zx}}{E_{zz}}\right)}^{-1}$$

where $E_{\zeta }$ is the Young modulus of the specimen evaluated in the ζ direction. Since the specimens were printed along seven different angles, seven different values of $G_{xz}$ can be evaluated from the tensile test data. The authors decided to use the value of $G_{xz}$ that minimized the difference between the experimental Young modulus and the theoretical ones $E_{\zeta ,th}$ computed by the inverse of Equation (9), as follows:

(10) $$E_{\zeta ,th}={\left(\frac{1}{E_{zz}}\;cos^{4}\theta +(\frac{1}{G_{xz}}-\frac{2\nu_{zx}}{E_{zz}})\;sin^{2}\theta \;cos^{2}\theta +\frac{1}{E_{xx}}\;sin^{4}\theta \right)}^{-1}$$

**Figure 5:**
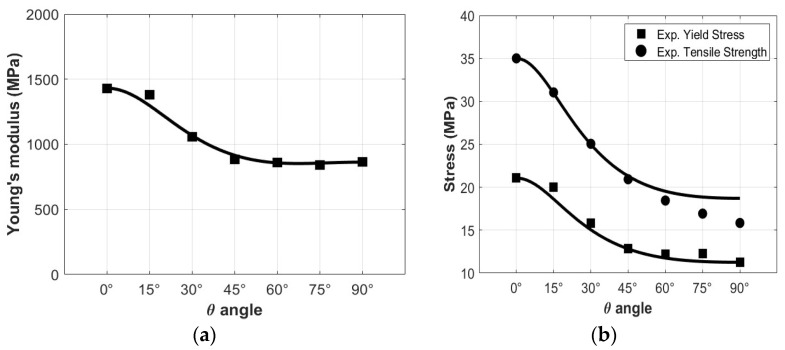
Comparison between experimental results (markers) and numerical predictions (continuous line) of Young’s modulus (**a**), yield stress and tensile ultimate strength (**b**) as a function of θ angle.

Figure 5a shows a comparison between experimental ($E_{\zeta }$) and theoretical ($E_{\zeta ,th}$) Young’s moduli calculated by Equation (10). Here, small differences were observed, thus proving that the proposed model can accurately evaluate Young’s moduli in various material directions. It is necessary to determine a condition that the yield stresses can be evaluated from. The non-linear elastic behavior of the material makes it difficult to determine an exact transition between the elastic and the plastic behavior, so an offset yield point of 0.2% plastic strain ($R_{p0.2}$) was used to evaluate the yield stress and the results are shown in Table 2. Once the elastic behavior was defined, the compliance matrix was filled with the parameters reported in Table 3.

Regarding yield analysis, given the specimens conformation, it is reasonable to adopt the plane stress state hypothesis. For the plane stress state, the yield criterion of Equation (7) becomes (neglecting the strain-rate effects):

(11) $$\left(G+H\right)\sigma_{xx}^{2}-2H\sigma_{xx}\sigma_{zz}+\left(H+F\right)\sigma_{zz}^{2}+2M\sigma_{xz}^{2}=f\left(\varepsilon_{eq}^{p}\right)$$

In this case, only four of Hill’s parameters needed to be defined. Hill’s parameters regarding normal yield stresses were calculated following the equations given in [20], while the one regarding shear yield stress was evaluated through a numerical optimization procedure. In particular, the distance of Hill’s yield locus from experimental yield points was minimized in order to find the best numerical fit for the *M* parameter. Hill’s parameters are reported in Table 4. Figure 5b reports comparisons between experimental results and numerical predictions of yield stresses (squared markers) and tensile ultimate strengths (circular markers), showing good agreement between the yield stresses. Focusing on ultimate tensile strengths, the continuous line was reproduced by scaling Hill’s parameters by the ratio between yield and ultimate tensile stress of the 0° specimen. The comparison shows that the isotropic evolution of Hill’s model captures the maximum loads reached for low-angle specimens, while specimens with higher angles show a different path, mainly due to their delamination behavior, as will be further discussed in Section 4.3.

**Figure 6:**
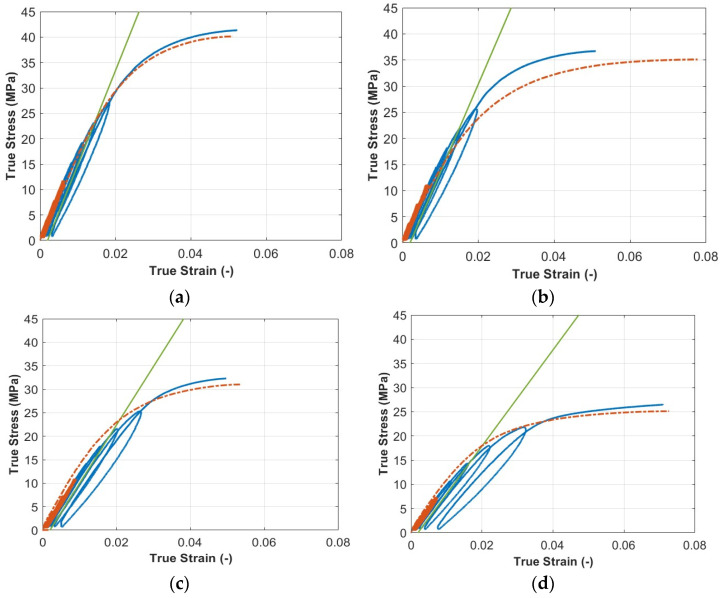

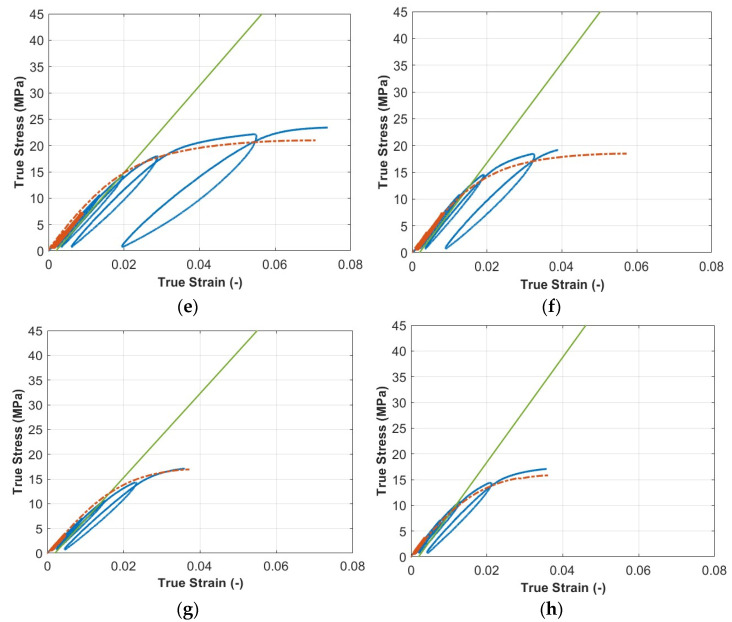
Stress–strain curves of cyclic tests conducted on the PL specimen (**a**), the 0° specimen (**b**), the 15° specimen (**c**), the 30° specimen (**d**), the 45° specimen (**e**), the 60° specimen (**f**), the 75° specimen (**g**), and the 90° specimen (**h**). Orange dashed curves represent monotonic stress–strain curves, while orange continuous lines represent the portion of the cyclic curve that was considered for Young’s modulus evaluation. Green lines are those used for the $R_{p0.2}$ yield stress evaluation.

In Figure 6, stress–strain curves of cyclic tests with corresponding monotonic tensile tests (conducted on a different batch) are reported. The curves are shown until the instability phase is reached, leaving out damage and failure. Cyclic tests were also useful to highlight similarities between the plastic behavior of printed polymeric materials and that of metallic ones. In fact, stress–strain curves show the hardening behavior of the material and that the approach to an instability phase at the maximum load can be achieved. In the early phases of cyclic tests, plastic deformation is already visible since the unloading phase is characterized by plastic hysteresis cycles. It can also be noted that the unloading phase is characterized by a non-linear elastic behavior. However, a linear elastic model can be used to represent the mechanical behavior of the material in the early loading phase. It is fair to say that the cyclic curves cannot be simply described by means of an elasto-plastic model since they show remarkable hysteresis cycles that are not expected from this type of model. As depicted in Figure 6, the monotonic and cyclic behaviors of the material are very similar, and the observable differences can be attributed to the normal dispersion observed between different batches.

It is worth noticing the differences between material properties obtained through the present work and the ones obtained by the manufacturer (reported in Table 1). Even though the adopted printing parameters are the same, the discrepancies in printing conditions should not be neglected. First of all, the printer used for the specimen fabrication was different, and other variables (such as the nozzle wear or filament stocking conditions) were not reported by the manufacturer, also because of the difficulties associated with evaluation. The present clarification intends to highlight how, even if the material is the same, the range of final conditions of printed components can be extremely wide. Indeed, in the authors’ opinion, it is more convenient to focus on the simplification of the identification process of printed material parameters instead of developing detailed material models with the intent of representing the effects of all the printing variables. In this way, a simpler procedure can be used to adapt to different printing conditions without directly considering their influence on material properties.

### 4.2. Numerical Model

**Figure 7:**
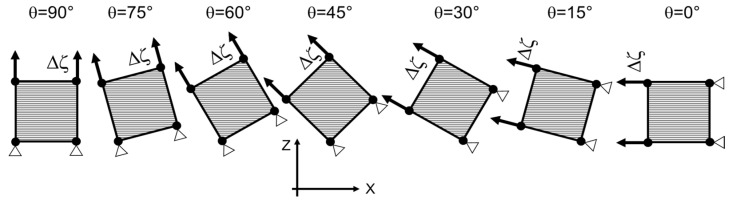
Numerical model configurations used for material model evaluation.

To analyze the results in the plastic flow phase, a numerical model was developed with seven four-node elements in the inclined plane stress formulation following the testing angle, as reported in Figure 7. It should be noted that the PL and 0° specimens were formally equal due to their printing configuration, but experimental results highlighted differences in Young’s moduli, yield stresses, and ultimate tensile strengths. For this reason, the authors decided to focus their modeling strategy on the 0° specimen only, since its condition represents the most conservational one from a mechanical design point of view. The software used for the simulations was LS-DYNA [29], which allows explicit non-linear finite element analyses to be performed and investigates the plastic behavior of the material. Among the many options offered by the software, the material model chosen was MAT_ANISOTROPIC_ELASTIC_PLASTIC (MAT157) since it allows anisotropy effects to be included (both in elastic and plastic stages), through the definition of an anisotropic stiffness matrix, Hill’s anisotropic yield criterion, and an isotropic hardening law. Two of the four nodes of each element were constrained in the translational motion in the testing direction, while a translational motion was imposed to the other two in order to reach the strain values of the experimental tests, as shown in Figure 7.

The anisotropic plastic deformation of the material is defined both by Hill’s criterion and by the associated hardening law. In this case, Voce’s isotropic hardening law with two parameters was chosen. Once the numerical model was implemented, it was used in a numerical optimization procedure by means of LS-OPT software [30]. The optimization procedure is typically conducted as a multi-objective optimization (MOO) problem; thus, each objective is not minimized at its fullest, but the solution represents a trade-off among different objectives [31]. The solution is found by changing the various parameters of the strain hardening law of the seven sub-models to reproduce experimental results with the best approximation possible. In this case, the difference between experimental and numerical load–displacement curves of the single specimen represents the single objective to be minimized and the weight assigned is evenly distributed among the different specimens. The mean square error (MSE) is the objective function used in the present work, while the chosen optimization algorithm is adaptive simulated annealing (ASA), which is automatically switched to the leap-frog algorithm for constrained optimization (LPCO) via the software [30].

**Figure 8:**
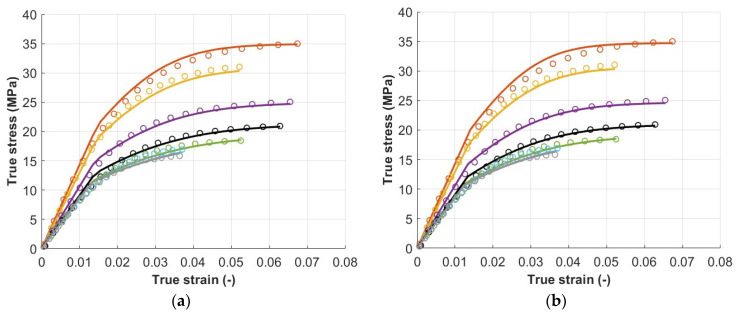
Comparison between experimental stress–strain curves (*σ_ζ_* and *ε_ζ_*) and numerical ones in the cases of Hill’s parameters directly identified from experimental data (**a**) and Hill’s optimized parameters (**b**). The MSE of the first case was 4.1 × 10^−3^ and that of the second case was 3.7 × 10^−3^.

The results of the optimization procedure can be observed in terms of the comparison between experimental and numerical true-stress–true-strain curves in the test direction (*σ_ζ_* and *ε_ζ_*) presented in Figure 8. Stress–strain curves were represented up to the maximum load since the model was not intended to reproduce instability and failure. The tensile behavior of the printed material was satisfactorily described by the model presented until the approach to the instability phase, where damage mechanisms and failure led to the apparent decrease in stress. Figure 8a shows the results obtained by fixing Hill’s parameters to the values directly identified from experimental data, while Figure 8b shows the results found with the optimization procedure that includes the optimization of Hill’s parameters, as presented in Table 5 and Table 6, respectively. The different optimization procedures led to very similar results, thus suggesting that the former is sufficient to capture material plastic behavior. Voce’s hardening law proves to be quite accurate in describing the plastic behavior of the material, and its combination with Hill’s yield criterion is able to capture the different stress evolution with respect to the various material directions.

### 4.3. Fracture Behavior

**Figure 9:**
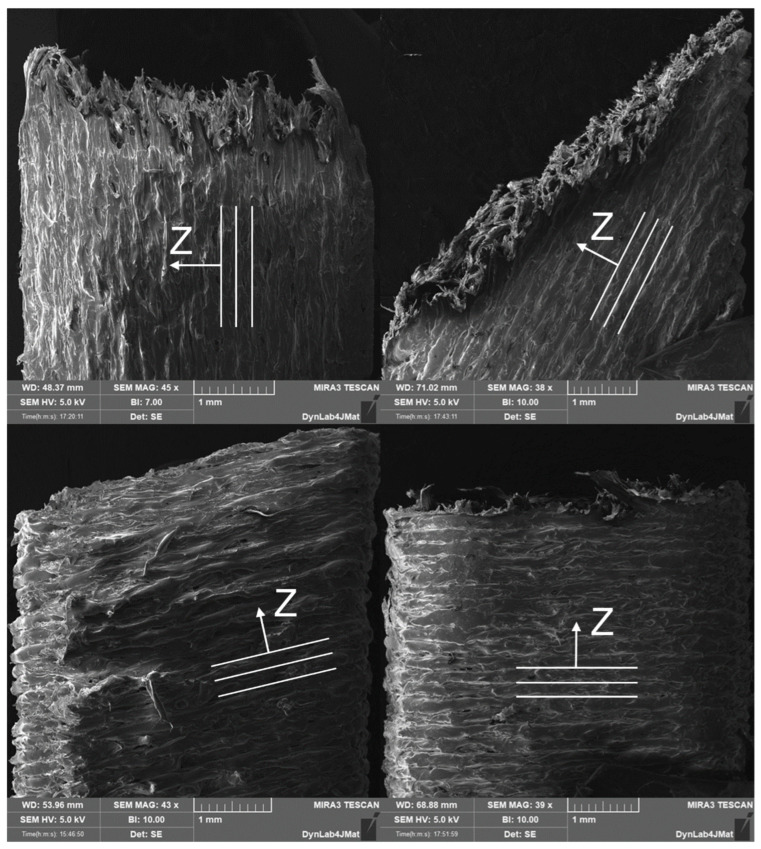
Electron microscope scans of fractured specimens. Broken surfaces of PL specimens (**top left**), the 30° specimen (**top right**), the 60° specimen (**bottom left**), and the 90° specimen (**bottom right**).

Experimental tensile tests showed a transition from intra-layer fracture (inside the layers) to inter-layer fracture (between layers), passing from low to high θ angles, as also highlighted in [17,18]. As shown in Figure 9, three different fracture modes can be identified: the first is characterized by high plastic deformation in the test direction, the second is denoted by shear failure at 45°, and the last one is characterized by layer delamination. Video analysis of tensile tests shows that PL and 0° specimens are prone to fail by the first failure mode; 15° and 30° specimens represent a transition between the first and the second; the 45° specimen represents the condition in which the last two modes coincide; and 60°, 75°, and 90° specimens are prone to fail by the third failure mode. The trend is also observable in Figure 5b where these last specimens show an early reach of the maximum load with respect to the others, moving away from the predicted behavior calculated with the scaling of Hill’s parameters via the 0° ratio between the specimen yield and the ultimate stress. However, if the fracture behavior can be simply described from a strict morphological point of view, the same cannot be said for the strain percentage at which the fracture occurs. In fact, experimental evidence shows that, in some cases, 30° and 45° specimens reach the highest strains at fracture, even overcoming PL and 0° specimens, while 15° values are very dispersed.

## 5. Summary and Conclusions

Given the growing expansion of fiber-reinforced polymeric materials to be used in FDM additive manufacturing, this technique is gradually evolving from simple aesthetics to small batches and pre-series production. As a consequence, this type of component needs to be designed and verified with respect to operational conditions. To conduct reliable design analyses, it is necessary to dispose of material models that are able to satisfactorily capture the mechanical behavior both in elastic and plastic fields, depending on whether the operational conditions will reach this range (i.e., in the case of structures designed for shock absorption and energy dissipation).

This study focuses on developing a methodology to calibrate an elasto-plastic model which takes into account printed material orthotropy concerning the elastic matrix, the yield criterion, and the plastic flow rule. The hardening law was also considered to be spatially orthotropic, as well as the yield criterion, but with an isotropic increase with respect to deformation (and possibly strain rate). This is also suggested by the strain-induced crystallization of the polymeric matrix that occurs regardless of the specimen orientation enhancing mechanical properties in the test direction. The identified material model is derived from a typical anisotropic model used for elasto-plastic metallic materials. With the intent to give the designer an effectively usable tool, the authors tried to limit the number and the complexity of experimental tests needed to calibrate the model as much as possible. In fact, in view of the largely variable mechanical characteristics of materials realized with the FDM technique with respect to the great number of technological parameters involved in the process, it is almost impossible to dispose of universally valid material models. It is more likely to obtain reliable results characterizing the material from time to time with respect to process parameters. It is thus possible to simultaneously produce the components and a small batch of dog bone specimens realized with different angles with respect to the printing plane for tensile tests. From the latter, it is possible to obtain information for the identification of five parameters that are necessary in order to define the elasticity matrix of a transversely isotropic material. This hypothesis, widely accepted in the FDM production field, identifies layer-by-layer material stratification as the main reason for anisotropy, neglecting possible differences obtained in the printing plane. The elastic field is then limited using Hill’s yield criterion for which four parameters are identified.

Eventually, by means of simple finite element models that reproduce standard test conditions combined with a numerical optimization algorithm, the hardening law parameters are identified. These parameters can operate in the whole plastic phase that characterizes the material behavior prior to instability and failure, whereby the Levi–Mises plastic flow rule, with volume conservation, is considered valid. This last aspect seems to introduce the strongest simplification hypothesis, since FDM materials, in addition to being anisotropic, are quite porous. However, to remove this hypothesis, it is necessary to experimentally measure all the components of the plastic strain increment tensor. This aspect should not be underestimated and analysis tools that are much more complex than those used in this work are required. In future works, microstructural experimentation and modeling (testing and modeling small material portions that are geometrically representative of the printing process) could be combined with the analyses proposed until now, thus allowing the relations between deformations to be precisely determined in different directions.

## Figures and Tables

**Table 1:** Summary of the datasheet properties of NylForce Carbon, as declared by the manufacturer, FiberForce.

Property (Units)	90° Specimen	PL Specimen
Tensile strength (MPa)	12.64	66.3
Elastic modulus (MPa)	1513	2758
Elongation at break (%)	2.0	6.7
Energy at break (J)	0.64	12.2
Density (g/cm^3^)	1.00	
Melting point (°C)	180	

Note: Tensile tests performed on specimens printed on Ultimaker 2+ with an Olsson Ruby nozzle temperature of 260 °C, a head bed temperature of 70 °C, a print speed of 40 mm/s, an infill percentage of 100%, and a raster angle of ±45°.

**Table 2:** Experimental results of tensile tests.

	0°	15°	30°	45°	60°	75°	90°
Young’s modulus $E_{\zeta }$ (MPa)	1431	1379	1057	882	863	841	863
Offset yield strength 0.2% (MPa)	21.07	20.02	15.80	12.85	12.22	12.25	11.26

**Table 3:** Compliance matrix coefficients.

$E_{xx}=E_{yy}\;(\mathrm{GPa})$	$E_{zz}\;(\mathrm{GPa})$	$G_{xy}\;(\mathrm{GPa})$	$G_{xz}=G_{yz}\;(\mathrm{GPa})$	$\nu_{zx}=\nu_{zy}\;(\text{-})$	$\nu_{xy}\;(\text{-})$
1431	863	511	331	0.22	0.45

**Table 4:** Hill’s parameters derived from experimental results.

F (1/MPa^2^)	G (1/MPa^2^)	H (1/MPa^2^)	M (1/MPa^2^)
0.003944	0.003944	−0.001619	0.011039

**Table 5:** Voce’s parameters found from the simulation with Hill’s parameters directly identified from experimental data (as shown in Table 4).

SIGY (MPa)	Qr_1_ (MPa)	Cr_1_ (−)
1	0.66	5.75

**Table 6:** Hill’s and Voce’s parameters of the fully optimized simulation.

S_11_ (MPa)	S_22_ (MPa)	S_33_ (MPa)	S_12_ (MPa)
10.59	20.05	20.05	6.36
**SIGY (MPa)**		**Qr_1_ (MPa)**	**Cr_1_ (-)**
1		0.73	7.29

## Data Availability

Not applicable.
